# Medication Adherence in Chronic Older Patients: An Italian Observational Study Using Medication Adherence Report Scale (MARS-5I)

**DOI:** 10.3390/ijerph19095190

**Published:** 2022-04-25

**Authors:** Gloria Liquori, Aurora De Leo, Emanuele Di Simone, Sara Dionisi, Noemi Giannetta, Elvira Ganci, Sherly Pia Trainito, Giovanni Battista Orsi, Marco Di Muzio, Christian Napoli

**Affiliations:** 1 Department of Biomedicine and Prevention, Tor Vergata University of Rome, 00133 Rome, Italy; gloria.liquori@uniroma1.it (G.L.); aurora.deleo@uniroma1.it (A.D.L.); sara.dionisi@uniroma1.it (S.D.); 2 Nursing, Technical, Rehabilitation, Assistance and Research Direction, IRCCS Istituti Fisioterapici Ospitalieri—IFO, 00144 Rome, Italy; emanuele.disimone@uniroma1.it; 3 UniCamillus—Saint Camillus International University of Health and Medical Sciences, 00131 Rome, Italy; noemi.giannetta@uniroma1.it; 4 Department of Medical Sciences, University of Ferrara, 44121 Ferrara, Italy; elvira.ganci98@gmail.com; 5 Department of Clinical and Molecular Medicine, Sapienza University of Rome, 00185 Rome, Italy; sherlytrainito@icloud.com (S.P.T.); marco.dimuzio@uniroma1.it (M.D.M.); 6 Department of Public Health and Infectious Diseases, Sapienza University of Rome, 00185 Roma, Italy; giovanni.orsi@uniroma1.it; 7 Department of Surgical and Medical Sciences and Translational Medicine, Sapienza University of Rome, 00185 Rome, Italy

**Keywords:** medication adherence, MARS, polypharmacy, communication, chronic disease

## Abstract

Background: the world population is aging, and the prevalence of chronic diseases is increasing. Chronic diseases affect the quality of life of patients and contribute toward increased healthcare costs if patients do not adhere to treatment. This study defines the medication adherence levels of patients with chronic diseases. Methods: an observational cross-sectional study was carried out. Patients aged 65 years and older with chronic diseases were included in this study. The medication adherence report scale was used. Results: overall, 98 patients aged 65 years and older were included. The mean age of responders was 78.65 years. Study population: 71.43% were always adherent; 9.79% often adherent; 14.89% sometimes adherent; 3.87% rarely adherent; and 1% never adherent. The internal consistency of the MARS-5I was good: Cronbach’s alfa value of 0.77. Conclusions: the MARS-5I is an effective self-report instrument to measure the medication adherence of patients. However, further studies are needed to explore factors affecting medication adherence to avoid clinical consequences for patients and high healthcare costs for healthcare facilities. Healthcare communication could be improved to ensure better transitional care.

## 1. Introduction

The world population is aging, and the prevalence of chronic diseases is increasing, leading to new challenges for healthcare facilities and healthcare workers. Specifically, this epidemiological trend is contributing to increasing occurrences of polypharmacy, which is defined as the regular use of five or more drugs in older adult patients [1]. Indeed, chronic disease drugs are meant to improve the symptoms and quality of life of patients, as well as treat problems related to chronic disease [2]. For instance, psychotropic polypharmacy during the initial phase of cancer care is widely used to treat associated psychological distress and mental disorders, but this use can be associated with significantly increased healthcare resource utilization, emphasizing the importance of correct prescriptions and close surveillance [3].

In Italy, 40.9% of the population is affected by at least one chronic disease [4]. Among patients aged 75 years and older, comorbidity stands at 65.1% (56.8% among men and 70.7% among women) [4]. In the latest “OsMed” report for the year 2020, it emerged that 98% of Italy’s elderly population has had at least one pharmacological prescription and there is an average intake of 6.7 different drugs per patient [5].

Polypharmacy can be an important risk factor for patients, caregivers, and healthcare professionals who manage the therapeutic process. The most important risks associated with polypharmacy include prescription errors [6], drug interactions (also due to errors in using the wrong drugs, due to similarities at the level of naming) [7], and increased mortality [8]. Moreover, attention must be paid to the level of the patient’s adherence to therapy; which is defined by the World Health Organization as “the degree to which the person’s behavior corresponds with the agreed recommendations from a health care provider” [9].

Adherence to a pharmacological treatment includes the achievement of at least two different objectives: the correct intake, according to the prescribed modalities (drugs, timing, doses, mode of intake), as well as persistence (constancy and continuity in the intake of the prescribed drugs) [10].

Medication adherence can be influenced by both sociodemographic factors, including age, gender, level of education, functional status (individual’s ability to perform daily activities required to maintain health) [11], and by care-related factors, such as the lack of proper communication during the patient’s transitional care process or public health campaign [12,13]; moreover, the presence or absence of a caregiver can influence medication adherence. Patients over 65 years of age, living alone, and undergoing chronic therapies have poor disease control and show poor adherence to therapy [14,15].

Medication non-adherence is also influenced by hospitalization: in hospitals, the patient’s therapeutic plans may vary; after discharge, the patient already shows little adherence to therapy and is often readmitted to the hospital [16]. This is known as the revolving door effect: patients leave the hospital after the disease has been stabilized and return after a few weeks/months because they stop the correct treatment [17]. Therefore, in addition to the possible short- and long-term repercussions on the patient’s intellectual sphere and the risk of disease progression and symptoms exacerbation, the related rehospitalization represents significant direct and indirect social costs [18,19,20]. Patients who relapse cannot reintegrate into the working environment and run the risk of remaining dependent on their families. As a result, the quality of familial–social relationships, as well as the quality of life of the patient, deteriorates.

In this context, it seems crucial to study medication adherence, especially in older adults who live alone and who have previous hospitalizations, since, at the time of patient discharge and readmission to the hospital, therapy management may change, which also influences medication adherence [21].

Despite extensive research on adherence to treatment, doubts about its correct measurement persist [22]. There are many measurement instruments, each with its own highlights and weaknesses: none of them can be considered a gold standard [23]. The Medication Adherence Report Scale (MARS© Prof. Rob Horne), a self-report questionnaire, originally developed in English, is described as one of the faster instruments to administer to patients, is easier for nurses to assess [24], and it is an appropriate scale to measure medication adherence in patients with non-communicable or chronic diseases [25]. The purposes of the present study were to (i) evaluate the levels of adherence to polypharmacy in spatiotemporally-oriented hospitalized patients, aged 65 years and older, with chronic diseases; and (ii) evaluate the possible association of polypharmacy adherence to the sociodemographic characteristics of the enrolled sample.

## 2. Materials and Methods

### 2.1. Type of Research

A cross-sectional study was performed during the period September–October 2021 in a large teaching hospital in Rome (central Italy), by using pre-validated questionnaires distributed to a sample of patients. People were invited to voluntarily participate in the survey by self-completing the questionnaires.

### 2.2. Participants

The study population was selected according to a model of non-probabilistic convenience sampling and following the stated inclusion criteria: age ≥ 65 years; male and female patients with a diagnosis of chronic disease; patients who used 5 or more drugs regularly; patients who had no difficulties in space, time, and orientation; patients who had been admitted to the hospital in the previous calendar year (since a critical moment for measuring adherence is when, after a discharge with a prescribed therapy, the patient is readmitted to the hospital), and patients who were not assisted by a caregiver.

Patients who had not been admitted to the hospital in the previous calendar year; who were assisted daily or occasionally by a caregiver; who had cognitive impairment or progressive neurological pathology (Alzheimer’s, Parkinson’s); or who had oncological pathology, were excluded from this study.

### 2.3. Instrument

The questionnaire was divided into two sections. The first section was aimed at collecting personal and sociodemographic information (age, gender, level of education, daily number of medications).

The second section was represented by the MARS-5I rating scale (Medication Adherence Report Scale© Prof. Rob Horne), which was used to assess levels of medication adherence of the selected sample. This instrument was translated into the Italian language and validated to assess adherence to pharmacotherapy in patients with Crohn’s disease [26].

The original version of the scale was also validated in English, to study other non-communicable or chronic diseases, such as stroke, chronic obstructive pulmonary disease, or inflammatory bowel disease [24,25,27].

This scale is an easily applicable instrument regardless of the pathology investigated and the drug administered and provides a reliable, valid, standardized measure of the patient’s adherence to drug therapy. MARS-5I consists of 5 items describing non-adherent behavior to the prescribed therapy. The items are assessed on a Likert scale (1–5) by analyzing the frequency with which the investigated attitude is carried out, ranging from “always” to “never”. The final score of the scale can range from 5, which corresponds to lowest adherence, to 25 points, maximum adherence (Supplementary Materials). To assess the patients’ spatiotemporal orientation ability, three simple questions were asked, in which all affirmative responses led patients to be recruited into the study: what season of the year are we in? Where are we now? What city are we in?

The questionnaires were distributed personally, explaining the rationale of the study and providing complete information for proper completion. To adhere to the research project, each questionnaire included an attachment for informed consent, signed freely. At first, an accurate medical history was performed for each respondent to determine the criteria for inclusion and exclusion. After this first phase, the enrolled patient was asked to fill in the self-report annexes in strict confidentiality. Questions were clarified without providing any suggestions about the answers to be included in the questionnaire. The collected data were imported to an Excel^®^ spreadsheet.

### 2.4. Ethics

MARS-5I was carried out with the informed consent of those concerned; anonymity was maintained by Legislative Decree 101/2018 and subsequent amendments.

All subjects gave their informed consent for inclusion before they participated in the study. The study was conducted in accordance with the Declaration of Helsinki, and the protocol was approved by the Ethics Committee, “Prot.0849/2021”. All data were treated in an aggregate form.

### 2.5. Statistical Analysis

Statistical software IBM SPSS Statistics^®^, version 26, was used in the statistical procedure. Information about gender, age, number of medications per day, and education qualification were collected. All of this information was analyzed using the descriptive analysis through the absolute frequencies and percentages. With regard to continuous variables, such as age and number of medications per days, the authors used the measures of central tendency, such as mean and range (maximum and minimum value).

A total score of all items of MARS-5I was calculated with a sum score ranging from 5 to 25 for adherence. The internal consistency was assessed using Cronbach’s *α*: values above 0.6 were generally considered satisfactory [28]. According to the instrument’s developer, the influence of the participants’ characteristics on adherence was assessed using a univariate logistic regression model derived from MARS-5I scores: MARS score < 25, lower treatment adherence; MARS score = 25, perfect treatment adherence. The outcomes analyzed were correlated to the following independent variables with the MARS scale: gender, age, education, number of medications per day. Specifically, with regard to independent variables, authors applied the dichotomizations of the variables: for example, age was grouped into 65–75 years versus 76–85 years versus 86–95 years.

## 3. Results

### 3.1. Professional and Sociodemographic Characteristics of the Sample

Table 1 shows the characteristics of responders. Among 103 patient candidates for the study, five (4.85%) refused to participate. The MARS-5I was responded to by 98 patients, joining the inclusion criteria, and with chronic disease diagnoses. The mean age of the responders was 78.65 years (SD±: 8.34 years; range: 65–95), with 51.0% female, 49.0% male; 40.8% had complete basic school education. The mean number of prescribed drugs per patient was 5.66 (SD±: 0.873). As reported in the inclusion criteria, all participants were oriented in time and space.

### 3.2. Disease and Drugs

The majority of the sample participants had cardiologic diseases (*n* = 74; 75.5%); 15.3% had metabolic diseases, and only 9.2% had respiratory diseases. Table 2 shows the list of drugs taken by the sample participants.

### 3.3. Self-Reported Adherence

Scores on the MARS-5I scale ranged from 5 to 25. The mean score was 22.43 (SD±: 3.11). The study population was 71.43% always adherent; 9.79% often adherent; 14.89% sometimes adherent; 3.87% rarely adherent; and 1% never adherent. Table 3 shows the characteristics of responders on the reported adherence. Table 4 shows the description of the MARS-5I and its individual items.

Regarding chronic diseases, the mean score of adherences in cardiological patients was 22.32; in metabolic patients was 23.47; and in respiratory patients was 21.67.

There were 34 (34.7%) participants with a MARS-5I sum score of 25. The internal consistency of the MARS-5I was good, performing a Cronbach’s alfa value of 0.77 [28], detailing any item Cronbach’s *α* ranging from 0.713 to 0.779 (Table 5).

### 3.4. Incidence of Patients’ Characteristics on Reported Adherence

Table 6 shows the reported adherence results of the univariable analysis of the logistic regression used to assess the incidence of patients’ characteristics. Concerning the maximum adherence—no significant predictor factors were found.

## 4. Discussion

Medication adherence is a hot topic in scientific literature. Many articles explore the concept and analyze factors that could affect medication adherence and its consequences. It is already known that a low level of medication adherence has consequences for the health status of patients and healthcare facilities [29].

Our findings show that the sample was always adherent in 71.43%; often adherent in 9.79%; sometimes adherent in 14.89%; rarely adherent in 3.87%; and never adherent in 1%, reporting a high MARS-5I mean score of 22.43. Previous studies in other countries have also reported a high medication adherence, although different tools to assess medication adherence have been used [15,30].

Regarding the use of MARS-5, our results agree with the study of Stone et al. [24]; however, it should be noted that this study was conducted in a sample of 155 non-hospitalized patients in order to validate the use of MARS-5 in patients with inflammatory bowel disease—a disease not reported in our study [24]. We found a mean MARS-5I score for respiratory patients of 21.67, against the 23.5 in a larger study carried out among patients with chronic obstructive pulmonary disease; also in this case, the patients were not hospitalized [25].

In our study, 33 males vs. 31 females showed lower treatment adherence and 15 males vs. 19 females showed perfect treatment adherence; but the association between gender and medication adherence was not statistically significant. This is consistent with another study, where, after controlling possible confounding effects of other covariates, gender was not confirmed as a significant predictor of treatment adherence in a sample of hospitalized patients with cardiovascular diseases [15]. A lack of association was also found regarding the educational level in the hospitalized subject, as well as in our study [15]. On the contrary, other larger-scale studies have shown that education is an essential parameter in predicting which patients adhere to drug therapy; these study enrolled patients from community pharmacies [31] or non-hospitalized patients with asthma [32,33].

Regarding age, Llorca et al. reported that in elderly patients (with multiple diseases and multiple medications), there is an association among non-adherence, self-medication, and worse lifestyle [34]. In our results, patients under 75 appeared to adhere slightly more than patients over 75, but the difference was not significant. This is consistent with findings from a study by Montes de Oca et al., where medication adherence was not associated with age [35].

Although, in our study, there was no association between the number of medications per day and treatment adherence, an inverse correlation between increasing the number of drugs/medications per day and decreased adherence was demonstrated [36,37]. For this reason, in case of polypharmacy prescription, communication between healthcare staff and patients and knowledge on the drugs used are crucial factors for adherence. Healthcare professionals must implement a proper communication process, especially in care transition when the therapeutic plan is communicated to the patient. Patients and physicians should cooperate to ease any problems, to enhance medication adherence resulting from other factors [30]. Moreover, it was demonstrated that, after discharge from hospital, the home setting can be a suitable venue for medication review and education. Assisting patients via an electronic personal health record system may enhance a pharmacist’s ability (and contribution) to identify and resolve medication-related problems that may lead to rehospitalization [38].

Considering what has been reported in the literature, hospitalization appears to be a discriminating factor—for medication adherence—to chronic therapy. Several studies show that, following hospital discharge, patients do not take their prescribed chronic therapy correctly, from 7 days until 2 years after discharge [21,39,40].

It should be noted that we decided to exclude caregiver-assisted participants, due to the influence that this support could have on patients with complex medication regimens. Social support could modulate any patient’s difficulties in adhering to therapy. Moreover, the role of caregivers is complex, and if, on the one hand, this figure is associated with increased medication adherence, on the other hand, it should be noted that violence against caregivers of older adults is not uncommon [41].

Finally, our sample reported a mean number of prescribed drugs per patient of 5.66, lower than the data reported by the “OsMed” report for the year 2020, according to which, for 98% of Italy’s elderly population, there was an average intake of 6.7 different drugs per patient [5]. Typically, a treatment plan with more than five medications reflects the presence of comorbidities at the individual level, making patients more susceptible to changes in dose, dosage, and time of intake.

## 5. Conclusions

In recent decades, research has shown that medication adherence is a variable phenomenon and is complex to evaluate. People aged 65 and older are potentially at risk of poor or incorrect medication adherence, given the incidence of chronic diseases and consequent polypharmacy. In our survey, high mean levels of medication adherence were found, especially for cardiovascular therapy. No discriminating parameters were identified to predict high drug therapy adherence; therefore, further studies are needed. Correct communication between healthcare personnel and patients, in order to clarify healthcare messages and improve medication adherence, was confirmed [42]. Several studies have also shown that telehealth and E-health interventions could represent further elements in the management of drug therapy and in the implementation of adequate medication adherence in patients with chronic diseases [43,44]. Among other strategies regarding the improvement of medication adherence, health communication intervention proved to be a key interactive element tailored to patients [30,45,46].

To date, medication adherence evaluation is a prerequisite for successful drug treatment; however, attention must be paid to the barriers that hinder it. Healthcare providers have prime roles in removing these barriers and in promoting interventions to improve patient adherence.

## 6. Limitations of the Study

The authors are aware of the limitations of this study. First, participants were enrolled by a convenience sampling of hospitalized patients in Rome, Italy; thus, the generalizability of our findings to other setting is limited. Second, the small number of participants may have affected the association between factors and medication adherence. Further studies are needed with larger samples that would minimize this problem. Moreover, regarding the recruitment of patients admitted in the previous calendar year, we did not consider the timing after discharge, which would have influenced medication adherence. This factor must be considered in future studies.

Finally, we should note that some of the drug classes listed in the tables are indicated as use “as-needed” and may not be appropriately included as items for the evaluation of daily use of medications (e.g., bronchodilators). Therefore, attention must be paid when evaluating this type of therapy.

## Figures and Tables

**Table 1:** Characteristics of responders.

Variables	*n* (%)
Gender	
Female	50 (51)
Male	48 (49.0)
Age (mean; range)	
	78.65 (65–69)
Number of medications per days (mean; range)	
	5.66 (5–9)
Educational qualification	
Basic school education	40 (40.8)
Middle school education	30 (30.6)
High school diploma	21 (21.4)
Degree	7 (7.1)

Professional and sociodemographic characteristics of the sample.

**Table 2:** List of drugs taken by the sample participants.

Variable	Yes (%)	No (%)
Stomach coater	82 (83.7)	16 (16.3)
Anti-hypertensive	71 (72.4)	27 (27.6)
Diuretics	43 (43.9)	55 (56.1)
Antiplatelet drugs	43 (43.9)	55 (56.1)
Anti-cholesterol drugs	35 (35.7)	63 (64.3)
Beta blockers	26 (26.5)	72 (73.5)
Hypoglycemic therapy	17 (17.3)	81 (82.7)
Anticoagulant	16 (16.3)	82 (83.7)
Insulin	15 (15.3)	83 (84.7)
New oral antiplatelet drugs	12 (12.2)	86 (87.8)
Anti-inflammatory drugs	10 (10.2)	88 (89.8)
Bile acid	8 (8.2)	90 (91.8)
Drug for the prostate	8 (8.2)	90 (91.8)
Cortisone	8 (8.2)	90 (91.8)
Painkiller	8 (8.2)	90 (91.8)
Iron tables	8 (8.2)	90 (91.8)
Vitamin D	8 (8.2)	90 (91.8)
Antiarrhythmic medication	5 (5.1)	93 (94.9)
Antibiotic	5 (5.1)	93 (94.5)
ACE inhibitor therapy	4 (4.1)	94 (95.9)
Benzodiazepine	4 (4.1)	94 (95.9)
Supplement	3 (3.1)	95 (96.9)
Heparin	3 (3.1)	95 (96.9)
Digitalis	2 (2)	96 (98)
Broncho dilatator medicines	2 (2.0)	96 (98)

**Table 3:** Characteristics of responders on reported adherence.

Variable	*n* (%)
**M1: I forget to take them**	
Always	0
Often	8 (8.2)
Sometimes	24 (24.5)
Rarely	19 (19.4)
Never	47 (48)
**M2: I change the dosage**	
Always	0
Often	4 (4.1)
Sometimes	5 (5.1)
Rarely	7 (7.1)
Never	82 (83.7)
**M3: I stop taking them for a while**	
Always	0
Often	2 (2.0)
Sometimes	16 (16.3)
Rarely	9 (9.2)
Never	71 (72.4)
**M4: I decide to skip taking a dose**	
Always	0
Often	2 (2)
Sometimes	21 (21.4)
Rarely	10 (10.2)
Never	65 (66.3)
**M5: I take them in a lesser amount than indicated to me**	
Always	0
Often	3 (3.1)
Sometimes	7 (7.1)
Rarely	3 (3.1)
Never	85 (86.7)

Sample is for the majority adherent to the therapies.

**Table 4:** Descriptive statistics of individual items of MARS-5I.

Variable	Mean (SD±), *n* = 98	Median (IQR) *n* = 98	Range
M1: I forget to take them	4.07	4.00	2–5
M2: I change the dosage	4.70	5.00	2–5
M3: I stop taking them for a while	4.52	5.00	2–5
M4: I decide to skip taking a dose	4.41	5.00	2–5
M5: I take them in a lesser amount than indicated to me	4.73	5.00	2–5

Mean, median, and range of adherences referring to each item category.

**Table 5:** Internal consistency of the MARS-5I.

Variable	Cronbach’s Alpha Value
M1: I forget to take them	0.713
M2: I change the dosage	0.779
M3: I stop taking them for a while	0.720
M4: I decide to skip taking a dose	0.714
M5: I take them in a lesser amount than indicated to me	0.743

**Table 6:** Incidences of patients’ characteristics on reported adherence.

Independent Variable	*n*	OR (95% IC)	B	*p*
Gender (male vs. female)				
Lower treatment adherence *	33 vs. 31	0.299 (0.585–3.110)	0.299	0.483
Perfect treatment adherence **	15 vs. 19			
Age (65–75 vs. 76–85)				
Lower treatment adherence *	23 vs. 22	−0.533 (0.204–1.500)	−0.592	0.245
Perfect treatment adherence **	17 vs. 9			
Age (65–75 vs. 86–95)				
Lower treatment adherence *	23 vs. 19	0.570 (0.202–1.607)	−0.563	0.288
Perfect treatment adherence **	17 vs. 8			
Number of medications per day		1.089 (0.679–1.745)	0.085	0.724
Educational qualification (Basic school education vs. Middle school education)				
Lower treatment adherence *	29 vs. 19	1.526 (0.552–4.218)	0.423	0.415
Perfect treatment adherence **	11 vs. 11			
High school diploma		2.397 (0.796–7.217)	0.874	0.120
Lower treatment adherence *	29 vs. 11			
Perfect treatment adherence **	11 vs. 10			
Degree		1.055 (0.178–6.257)	0.053	0.953
Lower treatment adherence *	29 vs. 5			
Perfect treatment adherence **	11 vs. 2			
Chronic disease (cardiologic vs. metabolic)		0.813 (0.260–2.535)	−0.208	0.721
Lower treatment adherence *	48 vs. 9			
Perfect treatment adherence **	26 vs. 6			
Chronic disease (cardiologic vs. respiratory)		1.896 (0.367–9.796)	0.640	0.445
Lower treatment adherence *	48 vs. 7			
Perfect treatment adherence **	26 vs. 2			
Chronic disease (respiratory vs. metabolic)		0.429 (0.065–2.810)	−0.847	0.377
Lower treatment adherence *	7 vs. 9			
Perfect treatment adherence **	2 vs. 6			

* MARS-5I score < 25; ** MARS-5I score = 25.

## Data Availability

The datasets generated and analyzed for this study can be requested from the correspondent author.

## Supplementary Materials

The following supporting information can be downloaded at: https://www.mdpi.com/article/10.3390/ijerph19095190/s1.

## References

[1] Masnoon N., Shakib S., Kalisch-Ellett L., Caughey G.E. (2017). What is polypharmacy? A systematic review of definitions. BMC Geriatr..

[2] Santalucia P., Franchi C., Djade C.D., Tettamanti M., Pasina L., Corrao S., Salerno F., Marengoni A., Marcucci M., Nobili A. (2015). Gender difference in drug use in hospitalized elderly patients. Eur. J. Intern. Med..

[3] Aroke H.A., Vyas A.M., Buchanan A.L., Kogut S.J. (2019). Prevalence of Psychotropic Polypharmacy and Associated Healthcare Resource Utilization during Initial Phase of Care among Adults with Cancer in USA. Drugs-Real World Outcomes.

[4] Annuario Statistico Italiano (2021). Sanità e Salute. https://www.istat.it/storage/ASI/2021/capitoli/C04.pdf.

[5] Rapporto Nazionale Anno (2020). L’uso dei Farmaci in Italia. https://www.aifa.gov.it/documents/20142/1542390/Rapporto-OsMed-2020.pdf.

[6] Dionisi S., Di Simone E., Liquori G., De Leo A., Di Muzio M., Giannetta N. (2021). Medication errors’ causes analysis in home care setting: A systematic review. Public Health Nurs..

[7] Giannetta N., Dionisi S., Ricciardi F., Di Muzio F., Penna G., Diella G., Di Simone E., Di Muzio M. (2019). Look-alike, sound-alike drugs: Strategies for preventing medication errors. [Farmaci LASA: Strategie per la prevenzione dell’errore di terapia]. G. Ital. Farm. Clin..

[8] De Vincentis A., Gallo P., Finamore P., Pedone C., Costanzo L., Pasina L., Cortesi L., Nobili A., Mannucci P.M., Incalzi R.A. (2020). Potentially Inappropriate Medications, Drug–Drug Interactions, and Anticholinergic Burden in Elderly Hospitalized Patients: Does an Association Exist with Post-Discharge Health Outcomes?. Drugs Aging.

[9] De Geest S., Sabaté E. (2003). Adherence to Long-Term Therapies: Evidence for Action.

[10] Brown M.T., Bussell J., Dutta S., Davis K., Strong S., Mathew S. (2016). Medication Adherence: Truth and Consequences. Am. J. Med. Sci..

[11] Sampaio R., Azevedo L.F., Dias C.C., Lopes J.M.C. (2020). Non-Adherence to Pharmacotherapy: A Prospective Multicentre Study about Its Incidence and Its Causes Perceived by Chronic Pain Patients. Patient Prefer. Adherence.

[12] Sumikawa Y., Yamamoto-Mitani N. (2021). Transitional care during COVID-19 pandemic in Japan: Calls for new strategies to integrate traditional approaches with information and communication technologies. Biosci. Trends.

[13] Gallè F., Sabella E.A., Roma P., Da Molin G., Diella G., Montagna M.T., Ferracuti S., Liguori G., Orsi G.B., Napoli C. (2021). Acceptance of COVID-19 Vaccination in the Elderly: A Cross-Sectional Study in Southern Italy. Vaccines.

[14] Tierney M.C., Snow W.G., Charles J., Moineddin R., Kiss A. (2007). Neuropsychological Predictors of Self-Neglect in Cognitively Impaired Older People Who Live Alone. Am. J. Geriatr. Psychiatry.

[15] G/tsadik D., Berhane Y., Worku A. (2020). Adherence to Antihypertensive Treatment and Associated Factors in Central Ethiopia. Int. J. Hypertens..

[16] Newby L.K., LaPointe N.M.A., Chen A.Y., Kramer J.M., Hammill B.G., DeLong E.R., Muhlbaier L.H., Califf R.M. (2006). Long-Term Adherence to Evidence-Based Secondary Prevention Therapies in Coronary Artery Disease. Circulation.

[17] Agana D.F.G., Salemi J.L., Striley C.W. (2019). From primary care to the revolving door of hospital readmission: Relevance of Geoffrey Rose’s call for a population strategy. Prev. Med. Rep..

[18] Vasan A., Morgan J.W., Mitra N., Xu C., Long J.A., Asch D.A., Kangovi S. (2020). Effects of a standardized community health worker intervention on hospitalization among disadvantaged patients with multiple chronic conditions: A pooled analysis of three clinical trials. Health Serv. Res..

[19] Caggiano G., Napoli C., Coretti C., Lovero G., Scarafile G., De Giglio O., Montagna M.T. (2014). Mold contamination in a controlled hospital environment: A 3-year surveillance in southern Italy. BMC Infect. Dis..

[20] Pasquarella C., Veronesi L., Castiglia P., Liguori G., Montagna M.T., Napoli C., Rizzetto R., Torre I., Masia M.D., Di Onofrio V. (2010). Italian multicentre study on microbial environmental contamination in dental clinics: A pilot study. Sci. Total Environ..

[21] Ho P.M., Spertus J.A., Masoudi F.A., Reid K.J., Peterson E.D., Magid D.J., Krumholz H.M., Rumsfeld J.S. (2006). Impact of Medication Therapy Discontinuation on Mortality after Myocardial Infarction. Arch. Intern. Med..

[22] Piña I.L., Di Palo K.E., Brown M.T., Choudhry N.K., Cvengros J., Whalen D., Whitsel L.P., Johnson J. (2021). Medication adherence: Importance, issues and policy: A policy statement from the American Heart Association. Prog. Cardiovasc. Dis..

[23] Lavsa S.M., Holzworth A., Ansani N.T. (2011). Selection of a validated scale for measuring medication adherence. J. Am. Pharm. Assoc..

[24] Stone J., Shafer L.A., Graff L.A., Lix L., Witges K., Targownik L.E., Haviva C., Sexton K., Bernstein C.N. (2021). Utility of the MARS-5 in Assessing Medication Adherence in IBD. Inflamm. Bowel Dis..

[25] Tommelein E., Mehuys E., Van Tongelen I., Brusselle G., Boussery K. (2014). Accuracy of the Medication Adherence Report Scale (MARS-5) as a Quantitative Measure of Adherence to Inhalation Medication in Patients with COPD. Ann. Pharmacother..

[26] Scribano M.L., Caprioli F., Michielan A., Contaldo A., Privitera A.C., Bozzi R.M., Calabrese E., Castiglione F., Ciccaglione A.F., Fave G.D. (2019). Translation and initial validation of the Medication Adherence Report Scale (MARS) in Italian patients with Crohn’s Disease. Dig. Liver Dis..

[27] Lin C.Y., Ou H.T., Nikoobakht M., Broström A., Årestedt K., Pakpour A. (2018). Validation of the 5-Item Medication Adherence Report Scale in Older Stroke Patients in Iran. J. Cardiovasc. Nurs..

[28] Taber K.S. (2018). The Use of Cronbach’s Alpha When Developing and Reporting Research Instruments in Science Education. Res. Sci. Educ..

[29] Bekker C.L., Aslani P., Chen T.F. (2021). The use of medication adherence guidelines in medication taking behaviour research. Res. Soc. Adm. Pharm..

[30] Thangsuk P., Pinyopornpanish K., Jiraporncharoen W., Buawangpong N., Angkurawaranon C. (2021). Is the Association between Herbal Use and Blood-Pressure Control Mediated by Medication Adherence? A Cross-Sectional Study in Primary Care. Int. J. Environ. Res. Public Health.

[31] Mekonnen G.B., Gelayee D.A. (2020). Low Medication Knowledge and Adherence to Oral Chronic Medications among Patients Attending Community Pharmacies: A Cross-Sectional Study in a Low-Income Country. BioMed Res. Int..

[32] Myers L., Murray R.K. (2019). Overcoming Health Literacy Barriers to Improve Asthma Inhaler Therapy Adherence. Ann. Am. Thorac. Soc..

[33] Apter A.J., Wan F., Reisine S., Bender B., Rand C., Bogen D.K., Bennett I., Bryant-Stephens T., Roy J., Gonzalez R. (2013). The association of health literacy with adherence and outcomes in moderate-severe asthma. J. Allergy Clin. Immunol..

[34] Llorca C., Castell E.C., Casado J.R., Ramos P.D.L., Ayestarán J.C., Blanco A.C., Gil Guillén V., Baeza M.R. (2021). Factors Associated with Non-Adherence to Drugs in Patients with Chronic Diseases Who Go to Pharmacies in Spain. Int. J. Environ. Res. Public Health.

[35] De Oca M.M., Menezes A., Wehrmeister F.C., Varela M.V.L., Casas A., Ugalde L., Ramirez-Venegas A., Mendoza L., López A., Surmont F. (2017). Adherence to inhaled therapies of COPD patients from seven Latin American countries: The LASSYC study. PLoS ONE.

[36] Murphy M., Bennett K., Ryan S., Hughes C.M., Lavan A.H., Cadogan C.A. (2021). A systematic scoping review of interventions to optimise medication prescribing and adherence in older adults with cancer. Res. Soc. Adm. Pharm..

[37] Bekelman T.A., Sauder K.A., Rockette-Wagner B., Glueck D.H., Dabelea D. (2021). Sociodemographic Predictors of Adherence to National Diet and Physical Activity Guidelines at Age 5 Years: The Healthy Start Study. Am. J. Health Promot..

[38] Kogut S., Goldstein E., Charbonneau C., Jackson A., Patry G. (2014). Improving medication management after a hospitalization with pharmacist home visits and electronic personal health records: An observational study. Drug Health Patient Saf..

[39] Jackevicius C.A., Mamdani M., Tu J. (2002). Adherence with Statin Therapy in Elderly Patients with and without Acute Coronary Syndromes. J. Am. Med. Assoc..

[40] AlHewiti A. (2014). Adherence to Long-Term Therapies and Beliefs about Medications. Int. J. Fam. Med..

[41] Pinyopornpanish K., Wajatieng W., Niruttisai N., Buawangpong N., Nantsupawat N., Angkurawaranon C., Jiraporncharoen W. (2022). Violence against caregivers of older adults with chronic diseases is associated with caregiver burden and depression: A cross-sectional study. BMC Geriatr..

[42] Giannetta N., Dionisi S., Tonello M., Cappadona R., Di Muzio M., Di Simone E. (2021). Educational intervention to improve the safety medication process: A review using the GRADE approach. J. Pharm. Health Serv. Res..

[43] Bingham J.M., Black M., Anderson E.J., Li Y., Toselli N., Fox S., Martin J.R., Axon D.R., Silva-Almodóvar A. (2021). Impact of Telehealth Interventions on Medication Adherence for Patients with Type 2 Diabetes, Hypertension, and/or Dyslipidemia: A Systematic Review. Ann. Pharmacother..

[44] Dionisi S., Di Simone E., Alicastro G.M., Angelini S., Giannetta N., Iacorossi L., Di Muzio M. (2020). Nursing Summary: Designing a nursing section in the Electronic Health Record. Acta Biomed..

[45] Glanz K., Beck A.D., Bundy L., Primo S., Lynn M.J., Cleveland J., Wold J.A., Echt K.V. (2012). Impact of a Health Communication Intervention to Improve Glaucoma Treatment Adherence. Arch. Ophthalmol..

[46] Giannetta N., Dionisi S., Villa G., Cappadona R., Fabbian F., De Giorgi A., Manara D.F., Di Muzio M., Manfredini R., Di Simone E. (2021). Characteristics of registered clinical trials assessing strategies of medication errors prevention. An unusual cross-sectional analysis. Acta Biomed..

