# Supplementary material for: Medication Adherence in Chronic Older Patients: An Italian Observational Study Using Medication Adherence Report Scale (MARS-5I)
Source: Int J Environ Res Public Health. 2022 Apr 25;19(9):5190. doi: 10.3390/ijerph19095190 (PMC9100757; doi:10.3390/ijerph19095190)
Supplement: Supplementary file 1 [file ijerph-19-05190-s001.zip › ijerph-1670302-supplementary.pdf]

Supplementary Materials

How do you take your prescribed medications?

This questionnaire is intended to assess the degree of adherence to your prescribed medications.

Please take the time to answer the questions carefully and completely.

Listed below are statements made by some subjects about their use of their prescribed medications. For each of these statements, please make a check mark (X) in the box that best reflects your behavior.

	Some behaviors about taking your prescribed medications	Always	Often	Sometimes	Rarely	Never
M1	I forget to take them					
M2	I change the dosage					
M3	I stop taking them for a while					
M4	I decide to skip taking a dose					
M5	I take them in a lesser amount than indicated to me					
